# A Minimal PKPD Interaction Model for Evaluating Synergy Effects of Combined NSCLC Therapies

**DOI:** 10.3390/jcm9061832

**Published:** 2020-06-12

**Authors:** Clara Mihaela Ionescu, Maria Ghita, Dana Copot, Eric Derom, Dirk Verellen

**Affiliations:** 1 Research Group of Dynamical Systems and Control, Ghent University, Tech Lane Science Park 125, 9052 Ghent, Belgium; Maria.Ghita@UGent.be (M.G.); Dana.Copot@UGent.be (D.C.); 2 EEDT—Core Lab on Decision and Control, Flanders Make Consortium, Tech Lane Science Park 131, 9052 Ghent, Belgium; 3 Department of Automatic Control, Technical University of Cluj Napoca, Memorandumului 28, 400114 Cluj-Napoca, Romania; 4 Department of Respiratory Medicine, Ghent University Hospital, C. Heymanslaan 10, 9000 Gent, Belgium; Eric.Derom@UZGent.be; 5 Iridium Cancer Network—GZA Hospitals Sint Augustinus, Department of Medical Physics Radiotherapy, Oosterveldlaan 24, 2610 Wilrijk, Belgium; Dirk.Verellen@UAntwerpen.be; 6 Radiotherapy Department, Faculty of Medicine and Health Sciences, Antwerp University, Universiteitsplein 1, 2610 Wilrijk, Belgium

**Keywords:** mathematical modelling, lung cancer, mouse data, antiangiogenic therapy, immunotherapy, radiotherapy, variability, synergy, multiple therapy, anomalous diffusion, fractal kinetics

## Abstract

This paper introduces a mathematical compartmental formulation of dose-effect synergy modelling for multiple therapies in non small cell lung cancer (NSCLC): antiangiogenic, immuno- and radiotherapy. The model formulates the dose-effect relationship in a unified context, with tumor proliferating rates and necrotic tissue volume progression as a function of therapy management profiles. The model accounts for inter- and intra-response variability by using surface model response terms. Slow acting peripheral compartments such as fat and muscle for drug distribution are not modelled. This minimal pharmacokinetic-pharmacodynamic (PKPD) model is evaluated with reported data in mice from literature. A systematic analysis is performed by varying only radiotherapy profiles, while antiangiogenesis and immunotherapy are fixed to their initial profiles. Three radiotherapy protocols are selected from literature: (1) a single dose 5 Gy once weekly; (2) a dose of 5 Gy × 3 days followed by a 2 Gy × 3 days after two weeks and (3) a dose of 5 Gy + 2 × 0.075 Gy followed after two weeks by a 2 Gy + 2 × 0.075 Gy dose. A reduction of 28% in tumor end-volume after 30 days was observed in Protocol 2 when compared to Protocol 1. No changes in end-volume were observed between Protocol 2 and Protocol 3, this in agreement with other literature studies. Additional analysis on drug interaction suggested that higher synergy among drugs affects up to three-fold the tumor volume (increased synergy leads to significantly lower growth ratio and lower total tumor volume). Similarly, changes in patient response indicated that increased drug resistance leads to lower reduction rates of tumor volumes, with end-volume increased up to 25–30%. In conclusion, the proposed minimal PKPD model has physiological value and can be used to study therapy management protocols and is an aiding tool in the clinical decision making process. Although developed with data from mice studies, the model is scalable to NSCLC patients.

## 1. Introduction

Drug dosing optimality problem for patient well-being with minimal impact on both economic and social resources has been recently addressed through multidisciplinarity, as information technology and computational power have much improved over the past decennia. Computer assisted drug therapy and long-term management in cancer patients have increased the outcome towards a personalised approach per individual as the medical information systems can accurately address inter- and intra-patient variability in response to treatment. A condition thereof is the existence of models to allow prediction of dose-effect as a function of chosen therapy and management protocol. Ideally, these models are individualised, hence their structure must go beyond curve fitting based population models.

It has been shown that combination therapy in cancer leads to synergic effects with improved outcome [1,2]. The SBRT (stereotactic body radiotherapy) is prevalent in lung cancer treatment as it is most patient friendly in terms of side effects, while proving to have excellent results [3,4,5]. Combined with antiangiogenesis and immunotherapy, SBRT could have a significantly improved efficacy.

This paper introduces a minimal model for lung cancer tumor growth prediction as a function of multiple therapy profiles. Multi-drug therapy results in surface dose-response characteristics, requiring to revisit the Michael-Mentis relationship [6,7]. Hill curve dose-effect dynamic variability can account for inter- and intra-patient variability [6,7]. Yet more, beyond the assumption of homogeneity in drug diffusion pattern in the body, fractal kinetics successfully characterized anomalous diffusion as a result of tissue heterogeneity in several applications with pharmacological data [8,9]. Latent response in drug therapy was assumed to be the result of drug trapping, leading to more accurate describing time-response of bolus administration [10,11].

Continuous dosing strategies are an emerging pattern in cancer treatment, but they require a prediction model to be used in the optimal cost function (e.g., to maintain tumor volume at a minimal value) [12,13]. Such strategies are introduced in computer assisted optimal dosing individualised protocols, where model parameters are identified from available data.

Multiple animal tumor models have been used in the development of chemotherapeutics and targeted therapies [14]. Similar experimentation led the development of immunotherapies to establish targeting efficiencies, pharmacokinetics/pharmacodynamics, whether there is spatial heterogeneity to therapy delivery, and whether therapy presence is related to efficacy. In this study we aimed to introduce the mathematical model formulation allowing further analysis into multiple therapy and translation to lung cancer patient data. This is a minimal model based on mouse data from literature and serves as a tool for further research and integration into a computer assisted optimal cancer treatment methodology.

## 2. Materials and Methods

### 2.1. Antiangiogenesis Therapy

This therapy has been proven to be effective in inhibiting the vascularization of tumor cell environment, thereby reducing the growth ratio of tumor volume [15,16]. Compared to monotherapy, combination immunotherapies have proven to significantly improve patient outcome. Several strategies have been reviewed, with novel strategies focusing on combining anti-angiogenic agents with chemotherapy or immunotherapy [17].

A realistic tumor growth model defining the tumor dynamics and necrotic tissue part as a result of antiangiogenesis treatment has been proposed in [18]. The model starts from a minimal formulation of the tumor growth under Bevacizumab treatment, extended with the volume and dynamics of the necrotic part and the pharmacodynamics (PD) and mixed-order pharmacokinetics (PK) of the applied drug.

The model is described by:

(1) $$\begin{array}{cccc} {\dot{x}}_{1}= & \left(a-n_{a}\right)x_{1} & -b_{a}\frac{x_{1}x_{3}}{ED50_{a}+x_{3}}\\ {\dot{x}}_{2}= & n_{a}x_{1} & +b_{a}\frac{x_{1}x_{3}}{ED50_{a}+x_{3}}\\ {\dot{x}}_{3}= & -c_{a}\frac{x_{3}}{KB_{a}+x_{3}} & -b_{a}^{k}\frac{x_{1}x_{3}}{ED50_{a}+x_{3}} & +u_{a} \end{array}$$

where $x_{1}$ represents the proliferating tumor volume (mm${}^{3}$), $x_{2}$ is the necrotic tumor volume (mm${}^{3}$) and $x_{3}$ is the inhibitor serum level (mg/mL) with $u_{a}$ the inhibitor dose rate (mg·day/mL). In this model representation, *a* is the tumor growth rate, $n_{a}$ is the necrosis rate, $b_{a}$ is the reaction rate coefficient on the inhibitory effect and $c_{a}$ is the clearance rate on the Michaelis-Menten kinetics $\frac{x_{1}x_{3}}{ED50_{a}+x_{3}}$ (mm${}^{3}$/day). The variable $b_{a}^{k}$ is the product of variable $b_{a}$ (mg/mL) and rate *k* (mg·mm${}^{3}$/mL), which is used to normalize units to allow inhibitor rate input $u_{a}$ in the equation.

This model has been identified in mice for Bevacizumab therapy and values are given in Table 1. Other values identified for Pegylated Liposomal Doxorubicin are given in [19].

### 2.2. Immunotherapy

Several types of immunotherapy are used to treat cancer, including immune checkpoint inhibitors, T-cell transfer therapy, monoclonal antibodies, immune system modulators, to mention a few. For lung cancer, immune checkpoint inhibitors are used with major breakthrough results, as reported in [20,21], although in some specific cases this clinical outcome may be limited [22]. Immunotherapy may cause side-effects such as rash, diarrhea, fatigue, or more rarely, widespread inflammation which leads to secondary organ effects (cough and chest pains in lung cancer patients). Intravenous administration of immunotherapy is considered in this study and the treatment can be applied every day, week or month, in cycles, followed by a rest period to allow recovery and response to immunotherapy. Although the benefit from this novel therapeutic approach is undeniable, several open questions still remain unanswered, as summarized in [21].

A model-based assessment of the relationship between Nivolumab exposure and safety was performed for doses varying from 1 to 10 mg/kg every 2 weeks in non small cell lung cancer (NSCLC) patients [23]. A previously published PK model was used to analyse the exposure-response in patients treated with Nivolumab indicating that clearance of drug changes with time, suggesting mixing heterogeneity and drug trapping. Averaged concentrations of 32±19 ∗ 10${}^{-3}$ mg/mL gave efficacy between 15–18%, with clearance rates varying between 6–18 mL/h (median 11.6 mL/h).

Combinations of 3-drug versus 2-drug immunotherapy increased efficacy from 23% to 53% in NSCLC patients [24,25]. Pembrolizumab combined with antiangiogenesis therapy, usually Bevacizumab, it allowed to go to lower doses [26], with efficacy between 19–43%. In mice, combined therapy provided up to 50% reduction of growth slope in tumor volumes [27].

Recent studies with Pembrolizumab in clinical trials in patients revealed significant added value of model based approach to treatment profile management [28]. Concentration-time profiles were simulated using the established population pharmacokinetic model of Pembrolizumab based on 2993 subjects from five clinical trials across tumor types. Efficacy was analysed by evaluating projections of average concentration over the dosing interval (Cavg) and trough concentration (Cmin) at steady state. Established exposure-response relationships for Pembrolizumab over a 5-fold dose range support that clinical efficacy and safety of higher single dose profile would be similar to the lower fraction doses across tumor types. Model based simulation studies in clinical trials for a Nivolumab monotherapy flat-dosing regimen have been given in [29]. The authors report that the time-averaged steady state exposure and safety profile of Nivolumab 480 mg every 4 weeks are consistent with that of 3 mg/kg every 2 weeks across multiple tumor types.

Other model based simulation supported study presenting various flat doses with mg/kg regimens is given in [30]. A flat dosing is expected to shorten preparation time and improve ease of administration. Based on population PK modelling, established flat exposure–response relationships for efficacy and clinical safety, the benefit–risk profile of Nivolumab 240 mg every 2 weeks was comparable to 3 mg/kg every 2 weeks. Such model based analysis is highly relevant, as the quantitative clinical pharmacology approach provided evidence for regulatory decision-making on dose modification, obviating the need for an independent clinical study.

Interestingly, model based retrospective studies as reported in [31], indicated that a significant proportion of advanced NSCLC patients receive Pembrolizumab-based regimens with extended intervals or delays in routine clinical practice and with similar outcomes to those receiving treatment at label-specified 3-week intervals.

To summarize, there is evidence to support the need for a PKPD model of combined antiangiogenesis and immunotherapy and the expectation that such combination therapy models will have great added value to the community. As detailed in [20], combination therapy of Pembrolizumab and Nivolumab had significant benefit, the patient characteristic, safety and tolerance should be considered in treatment decision-making. In this context, the proposed model hereafter includes features such as drug synergy and patient response to treatment which can be adjusted for further analysis.

### 2.3. Stereotactic Body Radiotherapy (SBRT)

With the advancement of the changing landscape of lung cancer treatment, radiation therapy (RT) is the most used modality to address local-regional areas of tissue. RT is a well-known cancer treatment, with complex and varied toxic biological effects, wanted to be provoked in diseased cells, while simultaneously avoiding healthy cells [32,33]. Stereotactic body radiation therapy (SBRT) is an advanced form of hypofractionated RT that consists of precise delivery of higher radiation doses on precised tumor location [3,15,34,35]. Stereotactic radiation using a linear accelerator has become a popular treatment for lung cancer. SBRT is an unconventional external beam radiation therapy designed for very precise tumor localization and radiation delivery. In SBRT, hypofractionated doses are applied. The principle can be described as follows: high doses of irradiation are delivered in a single dose or a few treatment fractions, avoiding treating a volume outside of the tumor target [4].

The continually downward bending form of a cell survival can simply be fitted by a second-order polynomial (i.e., the linear-quadratic (LQ) model), with a zero order constant term to ensure positive limit at zero dose. Simple radio-biological mechanisms linked to this model, whereas the cell survival rate is given by [36]:

(2) $$\begin{array}{c} -ln\left(S\right)=\alpha D+\beta D^{2}\\ p\left(survival\right)=exp\left(-\alpha D-\beta D^{2}\right) \end{array}$$

where α and β are coefficients of the fitted linear-quadratic equation, *D* is the total dose and this simple LQ formula gives a better description of radiation response in the low-dose region (0–3 Gy). LQ survival curves are continuously bending with no straight portion either at low or high radiation doses. The shape (or ‘bendiness’) is determined by the ratio α/β. In the beginning, the use of the LQ model was conceptually linked to the target-cell hypothesis. However, there is increasing evidence that many late effects, and even some early effects, of radiation therapy are not directly related to simple killing of a defined population of target cells [36]. The most prevalent current view is that the LQ approach represents an approximate, pragmatic method for converting dose–time fractionation schedules into a biologically effective dose. The basic equation for the incomplete repair model for continuous irradiation is:

(3) $$E=\alpha D+\beta D^{2}g$$

where *E* is the level of effect, *D* is the total dose and *g* is a function of the duration of continuous exposure:

(4) $$g=2\frac{\mu t-1+exp(-\mu t)}{{\left(\mu t\right)}^{2}}$$

where $\mu =\frac{0.693}{T_{1/2}}$, with $T_{1/2}$ (hours) the half time recovery and *t* (min) duration of exposure. The ratio α/β=10 (Gy) is equivalent to the coefficients ratio in the fractionated form of the input:

(5) $$u_{r}=a_{0}+a_{1}D+a_{2}Dd+\ldots$$

with $a_{i}$ the fraction, *D* the total dose and *d* the dose per fraction.

For mouse lung tumor radiation, the ratio α/β∈(13,17) and the effect dose 50% is ED50∈(15,35) (Gy) for dose rates of (1,100) (cGy/min). The Equation (2) is a second order polynomial with inflexion point and limit values, i.e., a sigmoid curve. In practice, only a part of this sigmoid curve is captured with data, whereas the full form can be computed by the incomplete repair model 3 which delivers a Hill curve dose-effect relationship [36].

There is evidence to support the claim that combined RT and immunotherapy leads to improved treatment efficacy [32,37]. Conventional radiotherapy, in addition to its well-established tumoricidal effects, can also activate the host immune system. Radiation therapy modulates tumor phenotypes, enhances antigen presentation and tumor immunogenicity, increases production of cytokines and alters the tumor microenvironment, enabling destruction of the tumor by the immune system. Multisite SBRT followed by Pembrolizumab was well tolerated with acceptable toxicity and increased overall survival in several clinical studies in NSCLC patients [38,39,40]. However, increased treatment efficacy was observed generally when radiotherapy was combined with immunotherapy in other types of tumors [37,41].

A recent review of effects of both radiotherapy and immune-checkpoint inhibition is given in [42]. The authors observe a complex interplay with the innate and adaptive immune systems, and under certain circumstances, the effects of radiotherapy synergize with those of immune-checkpoint inhibition to augment the antitumor responses typically observed with either modality alone and improve clinical outcomes. However, the mechanisms by which radiotherapy and immune-checkpoint inhibitors synergistically modulate the immune response might also affect both the type and severity of treatment-related toxicities. This is a line of investigation yet to be pursued. Generally, radiation promotes the release of danger signals and chemokines that recruit inflammatory cells into the tumor microenvironment, including antigen-presenting cells that activate cytotoxic T-cell function. By contrast, radiation can attract immunosuppressive cells into the tumor microenvironment. Sometimes the antitumor effect of radiotherapy has been observed outside of the radiation field, known as the abscopal effect, as discussed in [43]. There, the authors highlight new mechanistic explanations for the success or failure of radiotherapy, and postulate how the combination of immune-modulation and radiation could tip the balance of the host immune response to promote cure. Furthermore, other studies indicate that PK uptake may modulate RT effects [44], where tissue heterogeneity due to tumor changes plays an important role.

### 2.4. Proposed Combined Therapy Minimal Model

**Figure 1:**
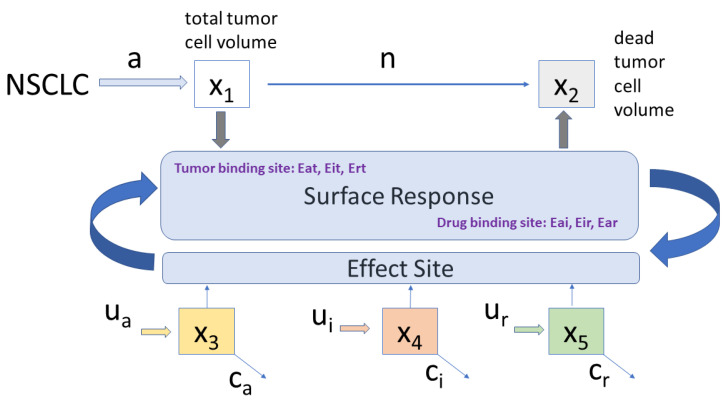
This is a schematic overview of the PKPD compartmental model.

Based on prior evidence summarized above, combining the three therapies in a single PKPD model representation is conditioned by adequate interpretation of synergistic effects and unit accommodation across the various inputs. Figure 1 depicts the proposed compartmental PKPD model.

The proposed minimal PKPD model is given by the set of equations:

(6) $$\begin{array}{cccc} {\dot{x}}_{1} & = & \left(a-n\right)x_{1} & -E\cdot x_{1}\\ {\dot{x}}_{2} & = & nx_{1} & +E\cdot x_{1}\\ {\dot{x}}_{3} & = & -c_{a}x_{3} & +u_{a}\\ {\dot{xe}}_{3} & = & -c_{a}xe_{3} & +Et_{a}\cdot x_{3}\\ {\dot{x}}_{4} & = & -c_{i}x_{4} & +u_{i}\\ {\dot{xe}}_{4} & = & -c_{i}xe_{4} & +Et_{i}\cdot x_{4}\\ {\dot{x}}_{5} & = & -c_{r}x_{5} & +u_{r}\\ {\dot{xe}}_{5} & = & -c_{r}xe_{5} & +Et_{r}\cdot x_{5} \end{array}$$

with $x_{i}$ expressed in mg/(mL·day). When combined with antiangiogenesis, immunotherapy increases the therapeutic effect with 25% [1,16,17,45] and up to 50% when combined with radiotherapy [15,34,35,46]. A linear combination of their synergic effect is considered for the tumor $Et_{all}$ and drug $Ed_{all}$ interactions:

(7) $$\begin{array}{ccc} Et_{all} & = & \frac{Eta+Eti+Etr}{3}\\ Ed_{all} & = & \frac{Eai+Ear+Eir}{3}\\ E & = & \frac{Et_{all}+Ed_{all}}{2} \end{array}$$

with Etx denoting the interaction between tumor cells and each drug, while Exy denotes interaction among drugs. When surface models are used to characterize synergic effects among drugs, the effect drug concentrations xe are normalized to their potency, i.e., to their corresponding half effect concentration $C_{50}$. The combined effects of two drugs $U_{A}$ and $U_{B}$ is considered as a new drug, and expressed as a Hill curve dose-response relationship 3D surface:

(8) $$Effect=\frac{I^{\gamma }}{1+I^{\gamma }}$$

with *I* denoting the interaction term:

(9) $$I=Un_{A}+Un_{B}+\sigma Un_{A}\cdot Un_{B}$$

with $Un_{A}=\frac{U_{A}}{C_{50A}}$ and $Un_{B}=\frac{U_{B}}{C_{50B}}$ the normalized drug effect concentrations and $C_{50}$ the concentrations at half effect 50%. The term γ denotes the sigmoidicity of the slope of the Hill surface, which indicates a patient drug responsiveness or drug resistance. The term σ denotes the amount of synergy present between the drugs. In the limits, when either one drug is used, the isobole response has values 0 to 1. If (8) is preceded by a maximum effect coefficient $E_{max}$, usually from 0% to 100% efficacy, then the effect is expressed in percent. A brief analysis of such surfaces is given in Appendix A.

The coefficients of the proposed model are listed in Table 2.

The initial tumor volume was fixed at 1000 mm${}^{3}$. The equivalent mass of the tumor from approximation formula 1 mm${}^{3}$ = $10^{-3}$ mg [36,46] was used to calibrate radiotherapy model parameters via a unit transformation from Gy ⟶ 1/mL. The reported tumor growth volumes are then relative to their initial value.

### 2.5. Protocols

The simulation time interval is 30 days, with a 1-day sample interval.

A control test was performed to check the tumor growth evolution without treatment.

The antiangiogenic (AG) therapy from [18] was used, with a 0.171 mg/mL dose applied once every week. The immunotherapy (IM) from [27] was used, with a 0.2 mg/mL dose applied once every week (corresponds to a full dose of 10 mg/kg). The doses for $u_{a}$ and $u_{i}$ were not modified throughout the study from these initial values.

The following RT protocols with values from [14] have been evaluated with the proposed PKPD model.

*Protocol 1:* A single dose of 5 Gy once weekly.

*Protocol 2:* A dose of 5 Gy × 3 days, followed by a 2 Gy × 3 days dose after two weeks.

*Protocol 3:* A dose of 5 Gy + 2 × 0.075 Gy, followed after 2 weeks by a 2 Gy + 2 × 0.075 Gy dose.

## 3. Results

**Figure 2:**
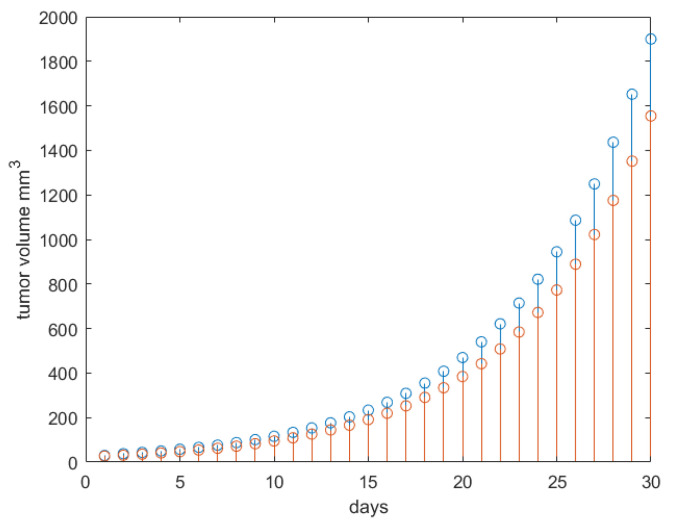
The total tumor growth volume (blue stems) and corresponding necrotic tumor volume (red stems) for no treatment.

The control test result is given in Figure 2. The result corresponds to that given in [18,34,46] and had the expected qualitatively clinical response [36].

**Figure 3:**
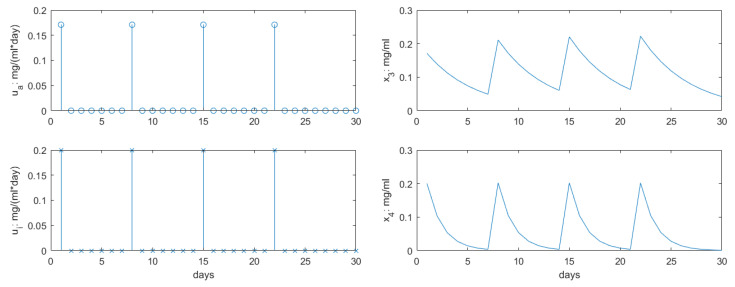
The dose rates and concentrations for the antiangiogenesis and immunotherapy drug profiles. These will remain fixed during the changes in radiotherapy profiles.

The profiles for inputs and concentrations for AG and IM drugs are given in Figure 3. These profiles correspond to literature studies as given in [18,27], respectively. In order to allow a systematic analysis of the proposed PKPD model, their values and time intervals will remain fixed, allowing to observe the effect of variation in radiotherapy profiles alone.

**Figure 4:**
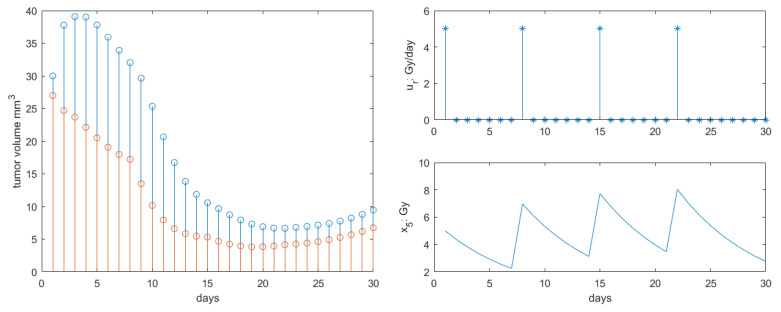
Results obtained for Protocol 1. **Left**: total tumor growth (blue stems) and necrotic tumor volume (red stems) response to therapy. **Right**: radiotherapy profile along with concentration profiles in body.

The results for Protocol 1 are depicted in Figure 4. As in [46], the single dose had effect as expected on the tumor growth response. Our profile response was accentuated compared to that in [46] as the additional antiangiogenesis and immunotherapy synergy was introduced. A 10-fold reduction in the tumor final volume at the end of the 30 days interval was observed compared to literature.

**Figure 5:**
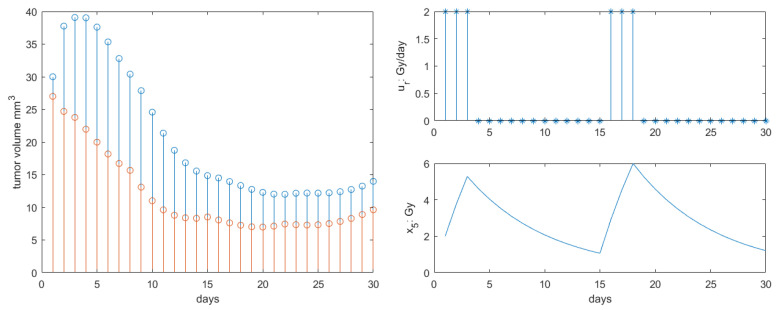
Results obtained for Protocol 2. **Left**: total tumor growth (blue stems) and necrotic tumor volume (red stems) response to therapy. **Right**: radiotherapy profile along with concentration profiles in body.

The results for Protocol 2 are depicted in Figure 5. The response was similar to that of Protocol 1, but the retardation of tumor growth was somewhat slower in dynamic response, with final volume at the end of the 30 days interval increased by 28% compared to Protocol 1. This is explained by lower concentration profiles in the first 15 days compared to Protocol 1, as observed in Figure 5—right. Similar observations were reported in [28], where protocols of single dose have similar clinical efficacy as fractionated lower dose protocols (but with same total intake).

**Figure 6:**
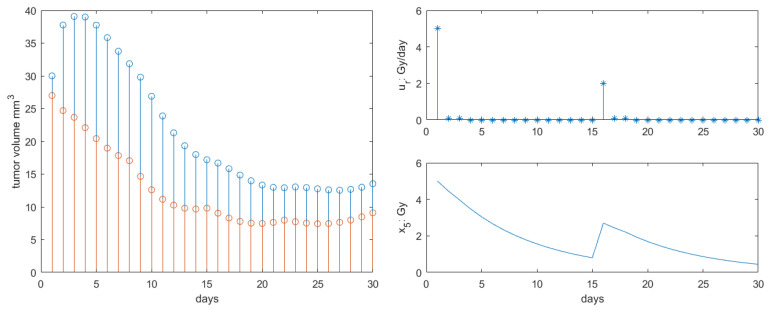
Results obtained for Protocol 3. **Left**: total tumor growth (blue stems) and necrotic tumor volume (red stems) response to therapy. **Right**: radiotherapy profile along with concentration profiles in body.

The results for Protocol 3 are depicted in Figure 6. There were no changes in the end tumor volume at the end of the 30 days interval when compared to Protocol 2. However, the retardation degree of tumor growth was somewhat decreased at the mid-interval as the concentration profile was lower than in Protocol 2, as observed from Figure 6—right.

**Figure 7:**
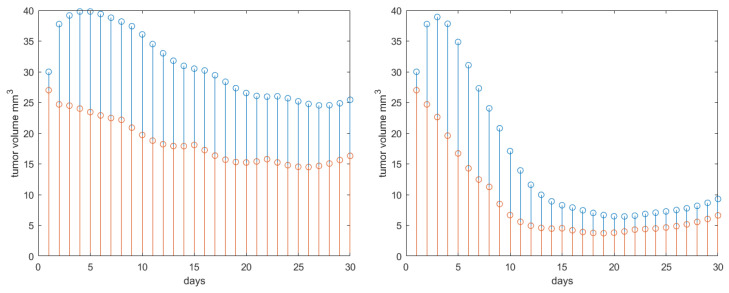
Results obtained for Protocol 3 in tumor growth volume with synergy degree varying from 1 (**left**) to 8 (**right**). Red stems denote the active tumor volume and blue stems the total tumor volume.

The systematic analysis is further deployed to capture the effect of drug synergy. For this, Protocol 3 is used. Varying synergy among drugs implies to vary among σ=1 (almost no synergy) and σ=8 (significantly high synergy). The simulation results are given in Figure 7 left and right, respectively. As expected, the tumor volume growth is not much reduced in the no synergy case, whereas in case of high synergy present, there is a three-fold reduction in final tumor volume at the end of the 30 days interval.

**Figure 8:**
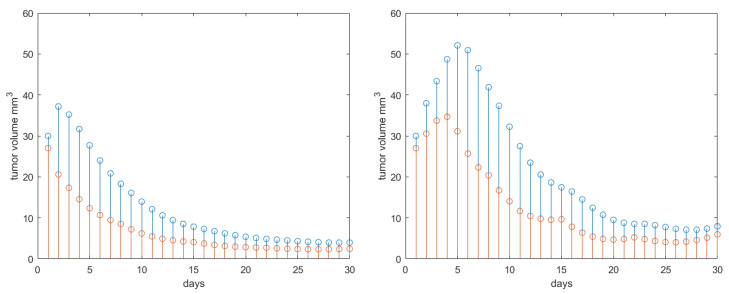
Results obtained for Protocol 3 in tumor growth volume with patient response degree varying from 1 (drug sensitive) to 8 (drug resistant). Red stems denote the active tumor volume and blue stems the total tumor volume.

To complete the systematic analysis of the features included by the proposed PKPD model, let us consider variations in patient response, i.e., a patient more responding to treatment if γ=1 and more resistant to treatment if γ=8. The drug synergy was set back to its nominal value σ=4. Protocol 3 is maintained for the radiotherapy profile. The results are given in Figure 8. As expected, if the patient response to treatment is higher, a pronounced reduction in tumor growth profile is observed. In presence of drug resistance, we observe an increase in tumor profiles along the 30 days interval with approximately 25–30%.

As a general remark, notice that the long tails observed in effects of RT (or SBRT) in weeks/months after treatment may be introduced in the model by changing the clearance value for $c_{r}$; here assumed to be 3 hours clearance in a day after treatment. This is of course limited for the purpose of the study to evaluate effects on tumor growth, but a more precise value assignment will give a more realistic long-term progression of tumor volume.

## 4. Discussion

The proposed PKPD model is minimal in its description as the number of compartments and phenomenological dynamics are described at macroscale pathway level. This provides a good overview of the essential features necessary to be introduced, such as effect sites, isobole surface models of interaction, degree of synergy among drugs and patient response to treatment (i.e., drug resistance). Despite its simplicity, the model was able to reproduce qualitatively tumor growth response to treatment as described in representative studies [14,18,36]. The modelled phenomena was based on physiological, chemical and molecular reactions described in literature [23,24,25] and general observations summarized in reviews [1,16,17,45]. When available, the numerical values introduced in the model parameters were assumed from literature, as referenced in Table 2. However, several parameters were not available, so the study introduced variability to assess their effect. The results suggest this is a fairly sensible model to be further calibrated on experimental data.

Although evaluated with mouse data, the model is directly scalable to diagnosed/treated NSCLC patients. The advantage of such a minimal PKPD model is that once calibrated for the patient at hand, hence addressing the personalised medicine concept, it can be used for evaluating various combination therapies. Reducing stronger drug doses will minimize side effects and incidence for later toxicity, thereby improving patient tolerance. Exposure–response (E–R) analyses of efficacy and safety can be used to optimize dosing regimens and establish a benefit–risk profile.

The toxicity and risk of radiation pneumonitis (RP) affecting the healthy tissue in proximity of tumor volume voxel during RT treatment are not included in this model. This is an important feature which should not be neglected [48]. The challenge for RT is to balance the high dose to targeted tumor and the lowest possible dose to organs at risk [49]. A local dose-effect model based on stretched exponential and power law is proposed in [50]. The model is successfully used to predict RP incidence in NSCLC treated with SBRT in patients. The model uses a normalized dose and a sigmoidal first order Hill curve formulation, which could be accommodated in the PKPD formulation of our model after some adequate transformation. The challenge to be addressed is that the model from [50] describes phenomenological effects at a different (mezo-)scale than our PKPD model proposed here.

Further yet more complex PKPD models are proposed in literature, describing effects of combination chemotherapy at micro-scale level [51]. Cooperative binding of transcription factors to various sites of a gene promoter or the formation of transcription factor multimers can account for nonlinearity in gene transcription. Inducing conformation changes in proteins, multisite phosphorylation provides an efficient way to control the catalytic activity of enzymes or the regulatory activity of transcription factors. The Hill function used to model such activity requires a delayed negative feedback, known as the Goodwin model [6]. This model can account for bistability oscillatory modes observed to arise from inhibitory Hill functions with delayed feedback. The implications of this are significant to model adverse effects with latent manifestation. This part is accounted by the power-law adaptation in regulatory networks and neural signaling pathways [52]. On the other hand, the assumption of a kinetic rate constant for the probability of events is not fully correct, for it has internal states of switching information transcription following fractal patterns [53,54]. The immediate utility of such observations are the justification of assuming variations in tissue uptake dynamics [9]. The transit times of drug release and absorption are in power-law form, which are the basis for introducing fractional calculus in modelling pharmacokinetics [8]. The concept of anomalous diffusion between compartments with different densities is introduced as a generalization of the classical PK modelling (e.g., central compartment is a fast acting one, while peripheral compartments such as muscle, fat, have slower dynamics). Effects of tissue heterogeneity are manifold in terms of dynamic analysis, an important one being the resident time that a drug remains bound to molecules before clearance from that compartment. Tissues with heterogeneous density have non-homogeneous drug distribution, hence drug trapping occurs [11]. This can be modelled in PK compartmental formulation by adding a memory term, able to characterize long tail dynamics of drug release. Again, the challenge to accommodate this in our model is the multi-scale pathway.

Tissue heterogeneity in tumor volume changes as a function of cancer cell proliferation, necrosis and washout, all functions of treatment profiles and drug interaction activity. As heterogeneity changes, assuming homogeneous drug distribution and uptake is not entirely realistic. Hence, a time dependent kinetic rate constant needs to be introduced. Furthermore, the patient response to drug therapy is changing in time as the patient may become drug resistant. This implies changes in the values for synergy degree σ and patient response γ. Heterogeneity and anomalous perfusion are observed in post-surgery lung cancer follow up studies [55]. As such, tissue heterogeneity in lungs has been assessed with maneuverless lung function tests in COPD patients [56,57]. The forced oscillation technique can be easily introduced to complement the follow up of NSCLC patients during their treatment as it does not require any forced maneuvers and it only requires tidal breathing measurement. Combined with CT scan information upon tumor size and local density variations, the lung function test can be used to provide correlations to the σ and γ model parameters from each individual measurement over time lapse of patient follow up.

Memory property in biological tissue response is modelled by fractional order derivatives [58], which can be implemented in efficient numerical computation forms [8]. Several applications of such descriptors are reviewed in [59]. Furthermore, the model from (2) is a combination of power law and exponential, which has been already proven mathematically to be fitted by the Mittag-Leffler function, which is a solution of the fractional derivative [8]. Such models have been shown to fit well the respiratory tissue properties in COPD [60,61] and other patients [62,63].

## 5. Conclusions

The paper introduces a minimal PKPD model for drug combination therapy analysis in NSCLC mice. The model is scalable to NSCLC patients. The drug synergic effects and patient response variability are introduced in the model by means of surface response models. The model agrees qualitatively with patho-physiological observations reported in literature. Tools such as PKPD models enable analysis into multi-drug therapy for NSCLC patients, while their results may reveal information to obviate the need for independent clinical studies. In view of time, cost and patient-related risks associated with unique emergency situations (e.g., pandemic outbreak), their relevance in regulatory decision-making on dose modification becomes crucial.

## Figures and Tables

**Table 1:** Identified averaged values from mice as reported in [18] for Bevacizumab antiangiogenic treatment.

Parameter	Name	Value	Units
*a*	tumor growth rate	0.4579	1/day
$b_{a}$	reaction rate	0.1685	1/day
$c_{a}$	clearance rate	0.1825	1/day
$n_{a}$	necrosis rate	0.1030	1/day
$b_{a}^{k}$	scaled inhibition rate	1.0839·$10^{-6}$	mg/(mL·day)
$KB_{a}$	Michaelis-Menten constant (inhibitor)	0.4409	mg/mL
$ED50_{a}$	half-effect concentration	50·10${}^{-6}$	mg/mL

**Table 2:** PKPD model coefficients values and units used in this study and the corresponding literature source. NA denotes source not available. Data from animal studies (mice).

Parameter	Name	Value	Units	Source
*a*	tumor growth rate	0.25	1/day	[18,46]
*n*	reaction rate	0.10	1/day	[18,46]
$c_{a}$	clearance rate Bevacizumab	0.1825	1/day	[18]
$c_{i}$	clearance rate Nivolumab	11.6/24	mL/day	[23]
$c_{r}$	clearance rate RT	3/24	1/day	[36]
$C50_{a}$	half-effect concentration Bevacizumab	0.44	mg/mL	[18]
$C50_{i}$	half-effect concentration Nivolumab	32·10${}^{-6}$	mg/mL	[23]
$C50_{r}$	half-effect concentration RT	20	Gy/day	[36]
$C50_{t}$	half-effect tumor growth	50	% mm${}^{3}$	[36,46]
$Emax_{a}$	max efficacy Bevacizumab	70	%	NA
$Emax_{i}$	max efficacy Nivolumab	43	%	[27]
$Emax_{r}$	max effect RT	50	%	[27,36]
γ	patient response/resistance to drug	2.5	(-)	[47]
σ	drug reaction (synergic)	4	(-)	[47]
*E*	combined effects (all)	calculated	1/day	NA
$u_{a}$	antiangiogenic drug dose rate	0.171	mg/(mL·day)	[18]
$u_{i}$	immunotherapy drug dose rate	0.20	mg/(mL·day)	[23,27]
$u_{r}$	radiotherapy dose rate	varies	mg/(mL·day)	[46]

**Figure A1:**
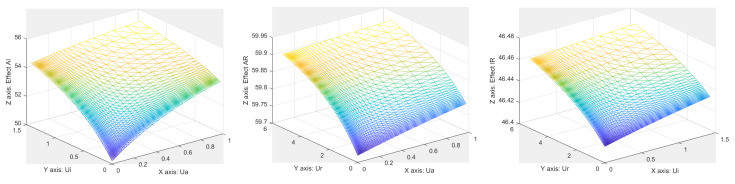
Surface responses obtained for σ=0.4 for each drug combination.

**Figure A2:**
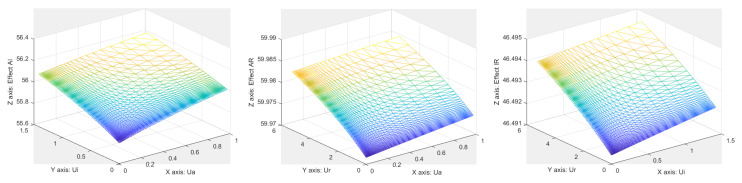
Surface responses obtained for σ=1 for each drug combination.

**Figure A3:**
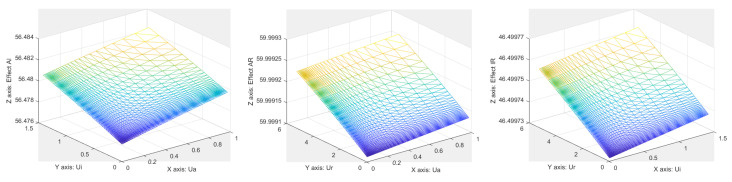
Surface responses obtained for σ=4 for each drug combination.

**Figure A4:**
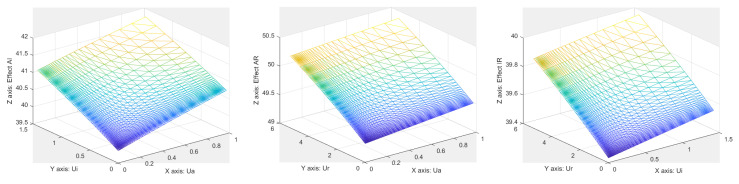
Surface responses obtained for γ=0.5 for each drug combination.

**Figure A5:**
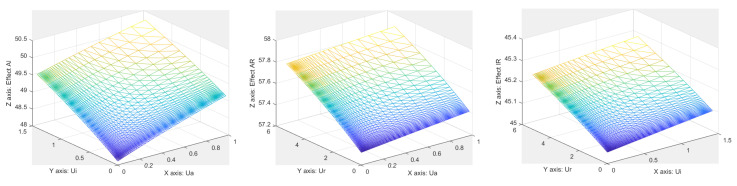
Surface responses obtained for γ=1 for each drug combination.

**Figure A6:**
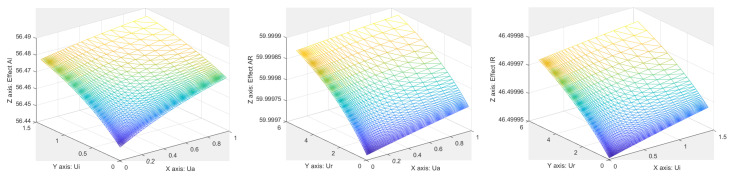
Surface responses obtained for γ=4 for each drug combination.

## Abbreviations

The following abbreviations are used in this manuscript:

AG	Antiangiogenic therapy
COPD	Chronic Obstructive Pulmonary Disease
IM	Immunotherapy
LQ	Linear-quadratic
NSCLC	Non small cell lung cancer
PD	Pharmacodynamic
PK	Pharmacokinetic
RP	Radiation Pneumonitis
RT	Radiotherapy
SBRT	Stereotactic Body Radiotherapy

## Appendix A

Study in the variability of σ and γ parameters for isobole surface response effects is given in [47]. Here we give a summary of variability effects for the three therapies combinations.

The patient response to drug therapy is fixed to γ=2.5, while the drug interaction synergy degree varies. The results are depicted in Figure A1 for σ=0.4, in Figure A2 for σ=1 and in Figure A3 for σ=4.

The drug synergy level is fixed to σ=1, while the patient response varies from 0.5<γ<4. The results are depicted in Figure A4 for γ=0.5, in Figure A5 for γ=1 and in Figure A6 for γ=4.

It can be observed that the surface area variability changes in agreement as expected from changes in the parameters.

## References

[1] Mennitto A., Huber V., Ratta R., Sepe P., de Braud F., Procopio G., Guadalupi V., Claps M., Stellato M., Daveri E. (2020). Angiogenesis and immunity in renal carcinoma: Can we turn an unhappy relationship into a happy marriage?. J. Clin. Med..

[2] Iafrate M., Fruhwirth G.O. (2020). How non-invasive in vivo cell tracking supports the development and translation of cancer immunotherapies. Front. Physiol..

[3] Liauw S., Connell P., Weichselbaum R. (2013). New paradigms and future challenges in radiation oncology: An update of biological targets and technology. Sci. Transl. Med..

[4] Roesch J., Andratschke N., Guckenberger M. (2014). SBRT in operable early stage lung cancer patients. Transl. Lung Cancer Res..

[5] de Jong E., Guckenberger M., Andratschke N., Dieckmann K., Milder M., Moller D.S., Nyeng T.B., Tanadini-Lang S., Lartigau E., Lacornerie T. (2020). Variation in current prescription practice of stereotactic body radiotherapy for peripherally located early stage non-small cell lung cancer: Recommendations for prescribing and recording according to the ACROP guideline and ICRU report 91. Radiother. Oncol..

[6] Gonze D., Abou-Jaoude W. (2013). The Goodwin model: Behind the Hill function. PLoS ONE.

[7] Goutelle S., Maurin M., Rougier F., Barbaut X., Bourguignon L., Ducher M., Maire P. (2008). The Hill equation: A review of its capabilities in pharmacological modelling. Fundam. Clin. Pharmacol..

[8] Sopasakis P., Sarimveis H., Macheras P., Dokoumetzidis A. (2018). Fractional calculus in pharmacokinetics. J. Pharmacokinet. Pharmacodyn..

[9] Weiss M. (2016). Comparison of distributed and compartmental models of drug disposition: Assessment of tissue uptake kinetics. J. Pharmacokinet. Pharmacodyn..

[10] Ionescu C.M., Copot D., De Keyser R. Modeling doxorubicin effect in various cancer therapies by means of fractional calculus. Proceedings of the 2016 American Control Conference (ACC).

[11] Copot D., Magin R., De Keyser R., Ionescu C.M. (2017). Data-driven modelling of drug tissue trapping using anomalous kinetics. Chaos Solitons Fractals.

[12] Sapi J., Kovacs L., Drexler D.A., Kocsis P., Gajari D., Sapi Z. (2015). Tumor volume estimation and quasi-continuous administration for most effective bevacizumab therapy. PLoS ONE.

[13] Kovacs L., Szeles A., Sapi J., Drexler D.A., Rudas I., Harmati I., Sapi Z. (2014). Model-based angiogenic inhibition of tumor growth using modern robust control method. Comput. Methods Programs Biomed..

[14] Cekanova M., Rathore K. (2014). Animal models and therapeutic molecular targets of cancer, utility and limitations. Drug Des. Dev. Ther..

[15] Kim D.W.N., Huamani J., Niermann K.J., Lee H., Geng L., Leavitt L.L., Baheza R.A., Jones C.C., Tumkur S., Yankeelov T.E. (2006). Noninvasive assessment of tumor vasculature response to radiation-mediated, vasculature-targeted therapy using quantified power Doppler Sonography. J. Ultrasound Med..

[16] Leone P., Buonavoglia A., Fasano R., Solimando A.G., De Re V., Cicco S., Vacca A., Racanelli V. (2019). Insights into the Regulation of Tumor Angiogenesis by Micro-RNAs. J. Clin. Med..

[17] Teleanu R.I., Chircov C., Grumezescu A.M., Teleanu D.M. (2020). Tumor Angiogenesis and Anti-Angiogenic Strategies for Cancer Treatment. J. Clin. Med..

[18] Drexler D.A., Sapi J., Kovacs L. (2017). Modeling of tumor growth incorporating the effects of necrosis and the effect of bevacizumab. Complexity.

[19] Drexler D.A., Ferenci T., Lovrics A., Kovacs L. Modeling of tumor growth incorporating the effect of pegylated liposomal doxorubicin. Proceedings of the 2019 IEEE 23rd International Conference on Intelligent Engineering Systems (INES).

[20] Almutairi A.R., Alkhatib N., Martin J., Babiker H.M., Garland L.L., McBride A., Abraham I. (2019). Comparative efficacy and safety of immunotherapies targeting the PD-1/PD-L1 pathway for previously treated advanced non-small cell lung cancer: A Bayesian network meta-analysis. Crit. Rev. Oncol. Hematol..

[21] Russo A.R., McCusker M.G., Scilla K.A., Arensmeyer K.E., Mehra R., Adamo V., Rolfo C., Naing A., Hajjar J. (2020). Immunotherapy in Lung Cancer: From a Minor God to the Olympus. Immunotherapy: Advances in Experimental Medicine and Biology.

[22] Pyfferoen L., Brabants E., Everaert C., De Cabooter N., Heyns K., Deswarte K., Vanheerswynghels M., De Prijck S., Waegemans G., Dullaers M. (2017). The transcriptome of lung tumor-infiltrating dendritic cells reveals a tumor-supporting phenotype and a microRNA signature with negative impact on clinical outcome. Oncoimmunology.

[23] Feng Y., Wang X., Bajaj G., Agrawal S., Bello A., Lestini B., Finckestein F.G., Park J.-S., Roy A. (2017). Nivolumab exposure-response analyses of efficacy and safety in previously treated squamous and non-squamous non-small cell lung cancer. Clin. Cancer Res..

[24] Nikanjam M., Patel H., Kurzrock R. (2017). Dosing immunotherapy combinations analysis of 3526 patients for toxicity and response patterns. Oncoimmunology.

[25] Nikanjam M., Liu S., Yang J., Kurzrock R. (2017). Dosing three-drug combinations that include targeted anti-cancer agents: Analysis of 37763 patients. Oncologist.

[26] Renn A., Burotto M., Rojas C. (2019). Immune checkpoint inhibitor dosing: Can we go lower without compromising clinical efficacy?. J. Glob. Oncol..

[27] Capasso A., Lang J., Pitts T.M., Jordan K.R., Lieu C.H., Davis S.L., Diamond J.R., Kopetz S., Barbee J., Peterson J. (2019). Characterization of immune responses to anti-PD-1 mono- and combination therapy in hematopoietic humanized mice implanted with tumor xenographs. J. Immunother. Cancer.

[28] Lala M., Li T.R., de Alwis D.P., Sinha V., Mayawala K., Yamamoto N., Siu L.L., Chartash E., Aboshady H., Jain L. (2020). A six-weekly dosing schedule for pembrolizumab in patients with cancer based on evaluation using modelling and simulation. Eur. J. Cancer.

[29] Long G.V., Tykodi S.S., Schneider J.G., Garbe C., Gravis G., Rashford M., Agrawal S., Grigoryeva E., Bello A., Roy A. (2018). Assessment of nivolumab exposure and clinical safety of 480 mg every 4 weeks flat dosing schedule in patients with cancer. Ann. Oncol..

[30] Zhao X., Suryawanshi S., Hruska M., Feng Y., Wang X., Shen J., Vezina H.E., McHenry M.B., Waxman I.M., Achanta A. (2017). Assessment of nivolumab benefit–risk profile of a 240-mg flat dose relative to a 3-mg/kg dosing regimen in patients with advanced tumors. Ann. Oncol..

[31] Sehgal K., Bulumulle A., Brody H., Gill R., Macherla S., Qilleri A., McDonald D.C., Cherry C.R., Shea M., Huberman M.S. (2020). Association of extended dosing intervals or delays in pembrolizumab-based regimens with survival outcomes in advanced non-small cell lung cancer. medRxiv.

[32] Deloch L., Deres A., Hartman J., Frey B., Fietkam R., Gaipl U.S. (2016). Modern RT concepts and the impact of radiation on immune activation. Front. Oncol..

[33] Abreu C.E.C.V., Ferreira P.P.R., de Moraes F.Y., Neves W.F.P., Godia R., Carvalho H.D.A. (2015). Stereotactic body radiotherapy in lung cancer: An update. J. Bras. Pneumol..

[34] Cho J., Kodym R., Seliounine S., Richardson J.A., Solberg T.D., Story M.D. (2010). High dose-per-fraction irradiation of limited lung volumes using an image-guided, highly focused irradiator: Simulating stereotactic body radiotherapy regimens in a small-animal model. Int. J. Radiat. Oncol. Biol. Phys..

[35] Du S., Lockamy V., Zhou L., Xue C., LeBlanc J., Glenn S., Shukla G., Yu Y., Dicker A.P., Leeper D.B. (2016). Stereotactic Body Radiation Therapy delivery in a genetically engineered mouse model of lung cancer. Int. J. Radiat. Oncol. Biol. Phys..

[36] Joiner M., van der Kogel A. (2009). Basic Clinical Radiobiology.

[37] Bernstein M.B., Krishnan S., Hodge J.W., Chang J.Y. (2016). Immunotherapy and stereotactic ablative radiotherapy (ISABR): A curative approach?. Nat. Rev. Clin. Oncol..

[38] Theelen W.S.M.E., Peulen H.M.U., Lalezari F., van der Noort V., de Vries J.F., Aerts J.G.J.V., Dumoulin D.W., Bahce I., Niemeijer A.-L.N., de Langen A.J. (2019). Effect of Pembrolizumab After Stereotactic Body Radiotherapy vs Pembrolizumab Alone on Tumor Response in Patients With Advanced Non-Small Cell Lung Cancer: Results of the PEMBRO-RT Phase 2 Randomized Clinical Trial. JAMA Oncol..

[39] Campbell A.M., Cai W.L., Burkhardt D., Gettinger S.N., Goldberg S.B., Amodio M., Kaech S., Krishnaswamy S., Decker R.H. (2019). Final Results of a Phase II Prospective Trial Evaluating the Combination of Stereotactic Body Radiotherapy (SBRT) with Concurrent Pembrolizumab in Patients with Metastatic Non-Small Cell Lung Cancer (NSCLC). Int. J. Radiat. Oncol. Biol. Phys..

[40] Schapira E., Hubbeling H., Yeap B.Y., Mehan W.A., Shaw A.T., Oh K., Gainor J.F., Shih H.A. (2018). Improved Overall Survival and Locoregional Disease Control With Concurrent PD-1 Pathway Inhibitors and Stereotactic Radiosurgery for Lung Cancer Patients With Brain Metastases. Int. J. Radiat. Oncol. Biol. Phys..

[41] Luke J.J., Lemons J.M.L., Karrison T.G., Pitroda S.P., Melotek J.M., Zha Y., Al-Hallaq H.A., Arina A., Khodarev N.N., Janisch L. (2018). Safety and Clinical Activity of Pembrolizumab and Multisite Stereotactic Body Radiotherapy in Patients With Advanced Solid Tumors. J. Clin. Oncol..

[42] Hwang W.L., Pike L.R.G., Royce T.J., Mahal B.A., Loeffler J.S. (2018). Safety of combining radiotherapy with immune-checkpoint inhibition. Nat. Rev. Clin. Oncol..

[43] Weichselbaum R.R., Liang H., Deng L., Fu Y.X. (2017). Radiotherapy and immunotherapy: A beneficial liaison?. Nat. Rev. Clin. Oncol..

[44] Chen Y.-J., Tsai T.-H., Wang L.-Y., Hsieh C.-H. (2017). Local radiotherapy affects the drug PK—Exploration of a neglected but significant uncertainty in lung cancer therapy. Technol. Cancer Res. Treat..

[45] Ren B., Rose J.B., Liu Y., Jaskular-Sztul R., Contreras C., Beck A., Chen H. (2019). Heterogeneity of Vascular Endothelial Cells, De Novo Arteriogenesis and Therapeutic Implications in Pancreatic Neuroendocrine Tumors. J. Clin. Med..

[46] Jin S.Z., Pan X.N., Wu N., Jin G.H., Liu S.Z. (2007). Whole-body low dose irradiation promotes the efficacy of conventional radiotherapy for cancer and possible mechanisms. Dose-Response.

[47] Ionescu C.M. (2018). A computationally efficient Hill curve adaptation strategy during continuous monitoring of dose-effect relation in anesthesia. Nonlinear Dyn..

[48] Dhont J., Vandemeulebroucke J., Burghelea M., Poels K., Depuydt T., Van Den Begin R., Jaudet C., Collen C., Engels B., Reynders T. (2018). The long- and short-term variability of breathing induced tumor motion in lung and liver over the course of a radiotherapy treatment. Radiother. Oncol..

[49] Vinh-Hung V., Leduc N., Verellen D., Verschraegen C., Dipasquale G., Nguyen N.P. (2019). The mean absolute dose deviation—A common metric for the evaluation of dose-volume histograms in radiation therapy. Med. Dosim..

[50] Selvaraj J., Lebesque J., Hope A., Guckenberger M., Werner-Wasik M., Peulen H., Giuliani M., Mantel F., Belderbos J., Grills I. (2019). Modelling radiation pneumonitis of pulmonary stereotactic body radiotherapy: The impact of a local dose-effect relationship for lung perfusion loss. Radiother. Oncol..

[51] Curtis L.T., van Berkel V.H., Frieboes H.B. (2018). Pharmacokinetic/pharmacodynamic modeling of combination chemotherapy for lung cancer. J. Theor. Biol..

[52] Drew P.J., Abbott L.F. (2006). Models and properties of power-law adaptation in neural systems. J. Neurophysiol..

[53] Liebovitch L.S., Scheurle D., Rusek M., Zochowski M. (2001). Fractal methods to analyze ion channel kinetics. Methods.

[54] Ionescu C.M. (2012). Phase Constancy in a Ladder Model of Neural Dynamics. IEEE Trans. Syst. Man Cybern. A Syst. Hum..

[55] Holvoet T., van Meerbeeck J.P., Van de Wiele C., Salhi B., Derom E. (2011). Quantitative perfusion scintigraphy or anatomic segment method in lung cancer resection. Lung Cancer.

[56] Ghita M., Copot D., Ghita M., Derom E., Ionescu C.M. (2019). Low frequency forced oscillation lung function test can distinguish dynamic tissue non-linearity in COPD patients. Front. Physiol..

[57] Copot D., De Keyser R., Derom E., Ionescu C.M. (2017). Structural changes in the COPD lung and related heterogeneity. PLoS ONE.

[58] Baleanu D., Fernandez A. (2018). On some new properties of fractional derivatives with Mittag-Leffler kernel. Commun. Nonlinear Sci. Numer. Simul..

[59] Ionescu C., Lopes A., Copot D., Machado Tenreiro J.A., Bates J.H.T. (2017). The role of fractional calculus in modeling biological phenomena: A review. Commun. Nonlinear Sci. Numer. Simul..

[60] Ionescu C., De Keyser R. (2009). Relations between fractional-order model parameters and lung pathology in chronic obstructive pulmonary disease. IEEE Trans. Biomed. Eng..

[61] Ionescu C., Derom E., De Keyser R. (2010). Assessment of respiratory mechanical properties with constant-phase models in healthy and COPD lungs. Comput. Methods Programs Biomed..

[62] Ionescu C.M. (2013). The Human Respiratory System: An Analysis of the Interplay between Anatomy, Structure, Breathing and Fractal Dynamics.

[63] Assadi I., Charef A., Copot D., De Keyser R., Bensouici T., Ionescu C.M. (2017). Evaluation of respiratory properties by means of fractional order models. Biomed. Signal Process. Control.

